# EBV-driven LMP1 and IFN-γ up-regulate PD-L1 in nasopharyngeal carcinoma: Implications for oncotargeted therapy

**DOI:** 10.18632/oncotarget.2608

**Published:** 2014-10-21

**Authors:** Wenfeng Fang, Jianwei Zhang, Shaodong Hong, Jianhua Zhan, Nan Chen, Tao Qin, Yanna Tang, Yaxiong Zhang, Shiyang Kang, Ting Zhou, Xuan Wu, Wenhua Liang, Zhihuang Hu, Yuxiang Ma, Yuanyuan Zhao, Ying Tian, Yunpeng Yang, Cong Xue, Yue Yan, Xue Hou, Peiyu Huang, Yan Huang, Hongyun Zhao, Li Zhang

**Affiliations:** ^1^ State Key laboratory of Oncology in South China, Department of Medical Oncology, Sun Yat-Sen University Cancer Center, Guangzhou, P. R. China; ^2^ Collaborative Innovation Center for Cancer Medicine, Sun Yat-sen University Cancer Center, Guangzhou, Guangdong, China; ^3^ Department of Oncology, the Sixth Affiliated Hospital of Sun Yat-sen University, Guangzhou, Guangdong, China; ^4^ Department of Oncology, the Fifth Affiliated Hospital of Sun Yat-sen University, Zhuhai, Guangdong, China

**Keywords:** Nasopharyngeal carcinoma (NPC), latent membrane protein 1 (LMP1), PD-L1, Epstein–Barr virus (EBV)

## Abstract

PD-L1 expression is a feature of Epstein-Barr virus (EBV) associated malignancies such as nasopharyngeal carcinoma (NPC). Here, we found that EBV-induced latent membrane protein 1 (LMP1) and IFN-γ pathways cooperate to regulate programmed cell death protein 1 ligand (PD-L1). Expression of PD-L1 was higher in EBV positive NPC cell lines compared with EBV negative cell lines. PD-L1 expression could be increased by exogenous and endogenous induction of LMP1 induced PD-L1. In agreement, expression of PD-L1 was suppressed by knocking down LMP1 in EBV positive cell lines. We further demonstrated that LMP1 up-regulated PD-L1 through STAT3, AP-1, and NF-κB pathways. Besides, IFN-γ was independent of but synergetic with LMP1 in up-regulating PD-L1 in NPC. Furthermore, we showed that PD-L1 was associated with worse disease-free survival in NPC patients. These results imply that blocking both the LMP1 oncogenic pathway and PD-1/PD-L1 checkpoints may be a promising therapeutic approach for EBV positive NPC patients.

## INTRODUCTION

Nasopharyngeal carcinoma (NPC) is one of the most common malignant tumors of head and neck in the Southeast Asia with an annual incidence of 15–50 cases per 100,000 persons [1]. Ninety-five percent of NPCs are characterized by undifferentiated or poorly differentiated squamous cell carcinoma. Radiotherapy or chemoradiotherapy is the main treatment methods for NPC [2, 3]. With the development of radiotherapy technique, the prognosis of early stage NPC has greatly been improved, with the 5-year local control rate and 5-year disease-free survival (DFS) rate of 95% and 77%, respectively [4]. However, the great potentiality of distant metastases remains the obstacles for survival improvement [1, 2]. Patients with advanced NPC have poor prognosis with a median survival time of only 5–11 months [2, 3]. Management of advanced NPC is therefore one of most challenging issues. Novel and effective therapy for NPC is urgently warranted.

Recently, tumor immune evasion is emerging as a hallmark of cancer [5]. The blockade of immune checkpoints has been the most promising approaches to activating antitumor immunity [6]. Cytotoxic T-lymphocyte-associated antigen 4 (CTLA4) antibodies were the first immunotherapeutic agents for melanoma with remarkable clinical response [7, 8]. Recently, several other immunomodulatory agents have shown great promise in clinical trials, especially anti-PD-1 and an-PD-L1 antibodies [9, 10]. More importantly, the treatment response of Nivolumab, an anti-PD1 antibody, is correlated with the expression of PD-L1 in a subset of tumors [10]. This discovery helps us to identify the right patients who will benefit from the immunomodulatory agents. However, the efficacy of such immune-targeted therapies in virus-associated malignancies remains unknown.

It is well known that NPC is a virus-driven malignancy [11, 12], which is characterized by prevailing Epstein-Barr virus (EBV) infection and the presence of immune infiltration around the cancer nests [13-15]. Activated immune cells such as cytotoxic tumor infiltrating lymphocytes (TILs) are important for eliminating residual cancer cells and monitoring recurrence. It has been reported that local infiltration of T-lymphocyte was a favorable indicator of survival in NPC patients [16]. However, many studies have indicated that NPC could escape the immune surveillance through different mechanisms [17, 18]. The diverse cellular mechanisms of immune evasion in NPC are largely undefined.

Recent studies showed that EBV-associated malignancies had high level of PD-L1, indicating that these tumors may be candidates for PD-1/PD-L1-directed therapies [19, 20]. However, the underlying mechanism of PD-L1 regulation in NPC with EBV infection is undetermined. In the present study, we aim to explore how EBV infection affects the expression of PD-L1 and its clinical significance in NPC patients.

## RESULTS

### PD-L1 expression in different human NPC cell lines

**Figure 1:**
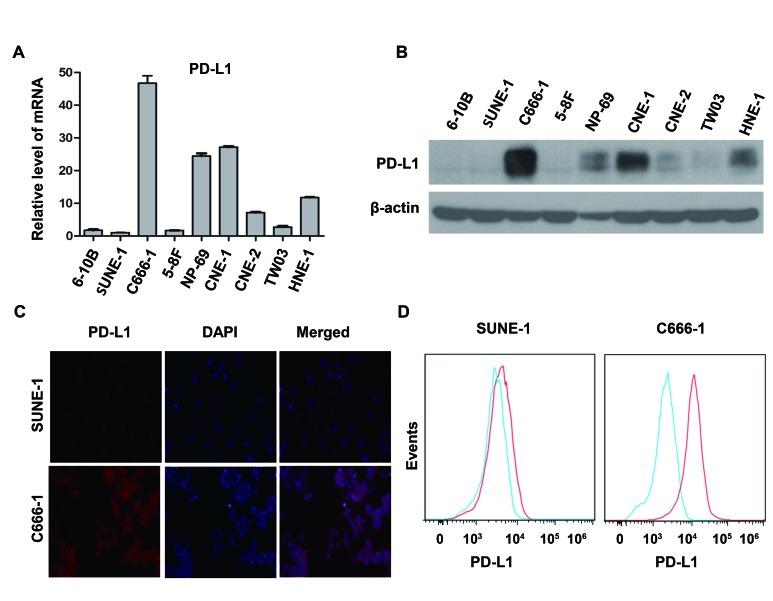
PD-L1 expression was associated with EBV infection in human nasopharyngeal carcinoma cell lines (A) The relative expression level of PD-L1 mRNA (detected by real time PCR method) in several common nasopharyngeal carcinoma cell lines (EBV-negative: CNE-1, CNE-2, SUNE-1, 5-8F, 6-10B, TWO3, and HNE-1; EBV-positive: C666-1) and an immortalized nasopharyngeal epithelial cell line (NP-69). The relative expression level of PD-L1 mRNA was normalized to that in SUNE-1 cell line. (B) The protein expression level of PD-L1 (detected by western blot) in different nasopharyngeal carcinoma cell lines and an immortalized nasopharyngeal epithelial cell line as described above. β-actin was used to verify equal loading. (C) The localization of PD-L1 (orange signal) in SUNE-1 and C666-1 cell lines shown by immunofluorescence counterstained with DAPI (blue signal). (D) Flow cytometric analysis of cell-surface PD-L1 expression in SUNE-1 and C666-1 cell lines (PD-L1, red line; isotype controls, blue line). All experiments were repeated at least three times. Representative data are shown.

To determine the expression of PD-L1 in NPC, we performed real time PCR and western blot to detect mRNA level and protein level of several common human NPC cell lines (EBV-negative: CNE-1, CNE-2, SUNE-1, 5-8F, 6-10B, TWO3 and HNE-1; EBV-positive: C666-1) and in an immortalized nasopharyngeal epithelial cell line (NP-69). Surprisingly, the relative expression level of PD-L1 mRNA in C666-1 cell line was remarkably higher than that in EBV-negative cell lines (Figure 1A), which was consistent with the protein level of PD-L1 in these cell lines (Figure 1B). Moreover, we employed immunofluorescence to locate PD-L1 in C666-1 cell line (with the highest PD-L1 expression) and SUNE-1 cell line (with very weak PD-L1 expression). Both of cell membrane and cytoplasm in the EBV-positive cell line (C666-1) showed strong PD-L1 signal (orange fluorescence), while the orange fluorescence signal of EBV-negative cell line (SUNE-1) was very weak (Figure 1C). The different level of PD-L1 expression in C666-1 and SUNE-1 was further confirmed by flow cytometry (Figure 1D).

### Enhanced expression of PD-L1 in constructed EBV-positive human NPC cell lines

**Figure 2:**
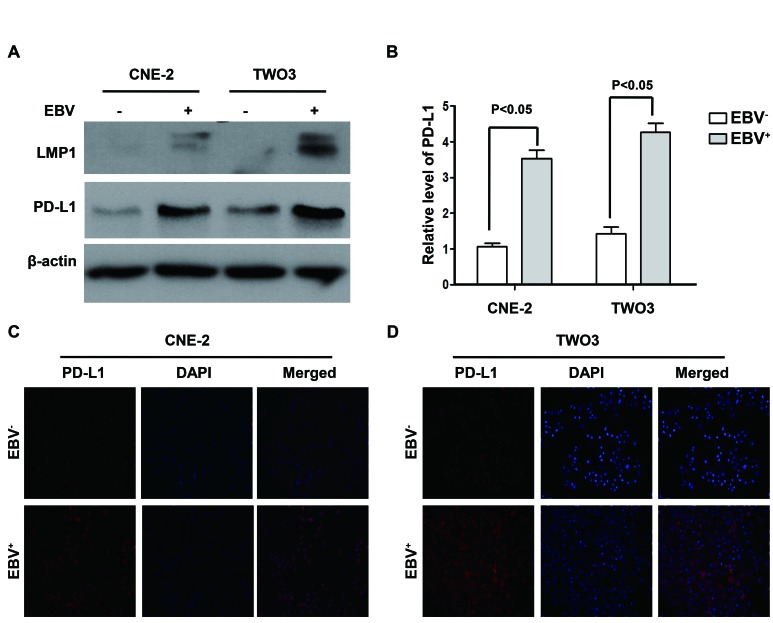
PD-L1 expression was induced by EBV infection in human nasopharyngeal carcinoma cell lines (A) The protein expression level of PD-L1 and LMP1 (detected by western blot) in the constructed EBV-positive (CNE-2-EBV^+^ and TWO3- EBV^+)^ and EBV-negative (CNE-2 and TWO3) parental cell lines. β-actin was used to verify equal loading. (B) Quantified protein expression level of PD-L1 in CNE-2, CNE-2- EBV^+^, TWO3 and TWO3- EBV^+^ cell lines using Quantity One software (Bio-Rad Laboratories, Hercules, CA). (C) The localization of PD-L1 (orange signal) in CNE-2 and CNE-2- EBV^+^ cell lines shown by immunofluorescence counterstained with DAPI (blue signal). (D) The localization of PD-L1 (orange signal) in TWO3 and TWO3- EBV^+^ cell lines shown by immunofluorescence counterstained with DAPI (blue signal). Representative data of three independent experiments are shown.

Two pairs of NPC cell lines (EBV-positive: CNE-2-EBV^+^ and TWO3-EBV^+^ vs EBV-negative: CNE-2 and TWO3) were constructed to determine whether PD-L1 expression in NPC cells was associated with EBV infection. The expression of PD-L1 at protein level in CNE-2-EBV^+^ and TWO3-EBV^+^ cell lines was significantly higher than that in their parental cell lines (CNE-2 and TWO3) (Figure 2A) and the quantification results are shown in Figure 2B. These results were further confirmed by flow cytometry method (supplementary Figure S1-A). Immunofluorescence showed the expression of PD-L1 was much more dense on the cell membrane and in the cytoplasm of CNE-2-EBV^+^ and TWO3-EBV^+^ cells than that of TWO3-EBV^−^ and CNE-2-EBV^−^ cells (Figure 2C and 2D).

### EBV infection up-regulated PD-L1 expression through LMP1 in human NPC cells

**Figure 3:**
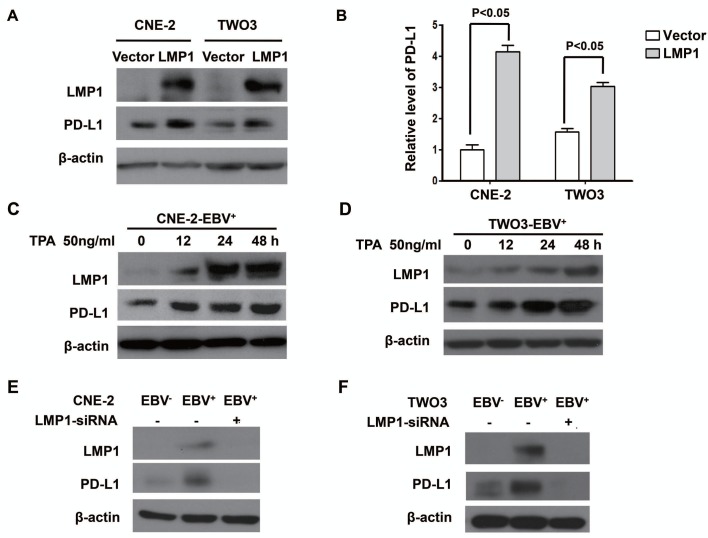
LMP1 mediated the up-regulation of PD-L1 expression in EBV-infected human NPC cells (A) The protein expression level of PD-L1 and LMP1 (detected by western blot) in CNE-2 and TWO-3 cell lines transiently transfected with control vector or LMP1 plasmids. (B) Quantified protein expression level of PD-L1 in CNE-2-vector, CNE-2-LMP1, TWO3-vector and TWO3-LMP1 cell lines using Quantity One software (Bio-Rad Laboratories, Hercules, CA). (C) The protein expression level of PD-L1 and LMP1 (detected by western blot) in CNE-2- EBV^+^ cell line treated with TPA (50 ng/ml) for 0, 12, 24 and 48 hours. (D) The protein expression level of PD-L1 and LMP1 (detected by western blot) in TWO3-EBV^+^ cell line treated with TPA (50 ng/ml) for 0, 12, 24 and 48 hours. (E) The protein expression level of PD-L1 and LMP1 (detected by Western blotting) in CNE-2-EBV^+^ cell lines and CNE-2-EBV^−^ cell lines after transfected with LMP1-siRNA or Mock-siRNA. (F) The protein expression level of PD-L1 and LMP1 (detected by Western blotting) in TWO3-EBV^+^ cell lines and TWO3-EBV^−^ cell lines after transfected with LMP1-siRNA or Mock-siRNA. All experiments were repeated at least three times and representative data are shown. β-actin was used to verify equal loading.

To clarify potential mechanisms of EBV-induced up-regulation of PD-L1 in NPC cells, we further determined whether LMP1 can regulate PD-L1 expression. First, we found that the expression of PD-L1 was positively correlated with LMP1 expression in EBV-infected NPC cell lines (CNE-2-EBV^+^ and TWO3-EBV^+^) (Figure. 2A and 2B). Second, NPC cells transfected with LMP1 (CNE-2-LMP1 and TWO3-LMP1) showed higher PD-L1 protein level compared with those transfected with control vectors (CNE-2-vector and TWO3-vector) (Figure 3A). The quantification analyses of Figure 3A are presented in Figure 3B. These results were further confirmed by flow cytometry method (supplementary Figure S1-B). Furthermore, we induced LMP1 expression by a chemical reagent 12-O-tetradecanoyl phorbol 13-acetate (TPA), which was reported to be an inducer of LMP1 in EBV positive cells through EBV replication [21]. As expected, the PD-L1 expression was increased following LMP1 induction by 50 ng/ml TPA treatment for 0, 12, 24 and 48 hours in both CNE-2-EBV^+^ and TWO3-EBV^+^ cells (Figure. 3C and 3D). In addition, we used siRNA to knock down the expression of LMP1 in CNE-2-EBV^+^ cells and TWO3-EBV^+^ cells. The PD-L1 expression was significantly decreased when LMP1 was knocked down by LMP1-siRNA (Figure 3E and 3F). These data imply that PD-L1 could be induced by EBV infection, which is mediated by LMP1.

### LMP1 induced PD-L1 expression through JAK3/STAT3, MAPKs/AP-1, and NF-κB pathways in human NPC cells

**Figure 4:**
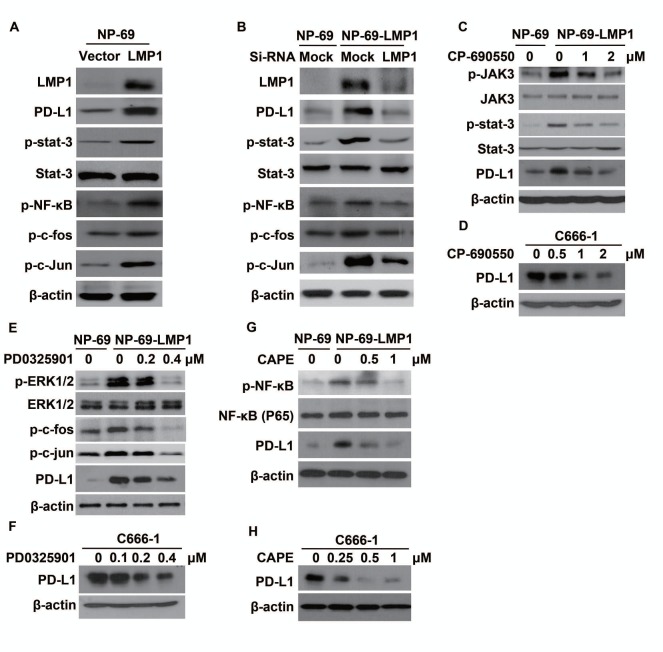
LMP1 induced PD-L1 expression through the downstream pathways involving JAK3/STAT3, AP-1 and NF-κB (A) The protein expression level of LMP1, PD-L1, p-STAT3, STAT3, p-NF-κB, p-c-fos and p-c-Jun (detected by western blot) in NP-69-vector and NP-69-LMP1 stable cell lines. (B) The protein expression level of LMP1, PD-L1, p-STAT3, STAT3, p-NF-κB, p-c-fos and p-c-Jun (detected by western blot) in NP-69-vector and NP-69-LMP1 stable cell lines after transfected with Mock-siRNA or LMP1-siRNA. (C) The protein expression alteration of p-JAK3, JAK-3, p-STAT3, STAT3, PD-L1 in NP-69-LMP1 or NP-69 cell lines treated with 0, 1, 2 μM CP-690550, a selective JAK3 inhibitor for 72 hours. (D) C666-1 cells were treated with 0, 0.5, 1.0, 2.0 μM CP-690550 for 72hours and the level of PD-L1 was detected by western blot. (E) The protein expression alteration of p-ERK1/2, ERK1/2, p-c-fos, p-c-Jun, PD-L1 in NP-69-LMP1 or NP-69 cell lines treated with 0, 0.2, 0.4 μM PD0325901, a selective MEKs inhibitor for 72hours. (F) C666-1 cells were treated with 0, 0.1, 0.2, and 0.4 μM PD0325901 for 72 hours and the level of PD-L1 was detected by western blot. (G) The protein expression alteration of p-NF-κB, NF-κB, PD-L1 in NP-69-LMP1 or NP-69 cell lines treated with 0, 0.5, and 1.0 μM Caffeic Acid Phenethyl Ester (CAPE), a selective p-NF-κB inhibitor for 72 hours. (H) C666-1 cells were treated with 0, 0.25, 0.5, 1.0 μM CAPE for 72 hours and the level of PD-L1 was detected by western blot. All experiments were repeated at least three times and representative data are shown. β-actin was used to verify equal loading.

Many studies found the activation of JAK3/STAT, AP-1 and p65/NF-κB mediates various downstream oncogenic effects of LMP1 [22-24]. To determine which pathway might be responsible for the up-regulation of PD-L1, we first tested the downstream pathways after LMP1 over-expression with western-blot. As expected, p-stat3, p-NF-κB, p-c-fos and p-c-Jun (two sub-units of AP-1) was activated by LMP1 (Figure 4A) and they were all reversed by LMP1-siRNA (Figure 4B). Next, we used inhibitors of JAK3, MEKs, or NF-κB to block the activation of downstream factors of LMP1. As shown in Figure 4C, selective inhibitor of JAK3 (CP-690550), can effectively inhibit the p-JAK3 and p-stat3 induced by LMP1, resulting in the decrease of PD-L1 expression. In addition, both PD0325901 (an inhibitor of MEKs) and Caffeic Acid Phenethyl Ester (an inhibitor of NF-ΚB) could effectively suppress LMP1-induced expression of PD-L1 in a dose-dependent manner (Figure 4E and 4G). The above-mentioned pathways were further confirmed in C666-1 with constitutive EBV infection (Figure 4D, 4F, and 4H). These results showed that LMP1 induced PD-L1 expression at least partly through JAK3/STAT3, MAPKs/AP-1 and p65/NF-κB pathways in human NPC cells.

### IFN-γ up-regulated PD-L1 expression in human NPC cells which was independent of but synergetic with EBV infection

**Figure 5:**
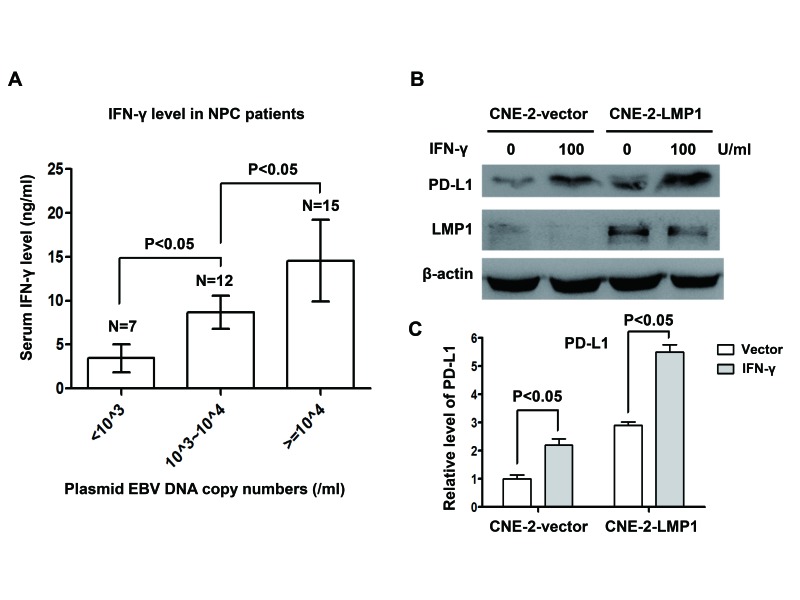
IFN-γ up-regulated PD-L1 expression in human nasopharyngeal carcinoma cells, which was independent of but synergetic with LMP1 (A) Serum IFN-γ level and EBV DNA copy numbers were measured in 34 NPC patients. Serum IFN-γ level was positively correlated with EBV burden. (B) The protein expression level of PD-L1 and LMP1 (detected by western blot) in CNE-2-vector and CNE-2-LMP1 stable cell lines treated with or without IFN-γ (100 U/ml) for 48 hours. β-actin was used to verify equal loading. (C) Quantified protein expression level of PD-L1 in CNE-2-vector and CNE-2-LMP1 cell lines using Quantity One software (Bio-Rad Laboratories, Hercules, CA) after IFN-γ treatment (100 U/ml) or not.

We analyzed plasma EBV DNA burden and serum IFN-γ level in 34 NPC patients to explore the relationship between EBV infection and IFN-γ. According the plasmid EBV DNA copy number, we divided the population into three groups including EBV DNA copy number less than 10^3^/ml group, 10^3^/ml~10^4^/ml and more than 10^4^/ml groups. We found that serum IFN-γ level increased along with increasing EBV burden (*P*<0.05, Figure 5A). In order to investigate whether EBV infection could directly induce the production of IFN-γ in NPC cells in vitro, we tested the level of IFN-γ in NPC cell lines. The results showed that no IFN-γ mRNA was detected in NPC cell lines both before and after EBV infection (supplementary Figure S2-A). Next, we found no IFN-γ was excreted into the culture medium of NPC cell lines before and after EBV infection (supplementary Figure S2-B). These results imply that the production of IFN-γ in NPC patients may be mediated by other cells after EBV infection, possibly by the infiltrating T lymphocytes. To determine whether IFN-γ could regulate PD-L1 expression and its relation with LMP1-mediated PD-L1 up-regulation, NPC stable cell lines translated with control vector and LMP1 (CNE-2-vector and CNE-2-LMP1) were treated with or without 100U/ml IFN-γ for 24 hours. We found that PD-L1 expression was up-regulated in both CNE-2-vector and CNE-2-LMP1 cells after IFN-γ treatment. However PD-L1 expression was much higher in CNE-2-LMP1 cells than in CNE-2-vector cells with IFN-γ treatment (Figure 5B and 5C). These results show that IFN-γ up-regulates PD-L1 expression in human NPC cells which is independent of but synergetic with LMP1.

### Disease-free survival of NPC patients was associated with PD-L1 expression in tumor tissues

**Figure 6:**
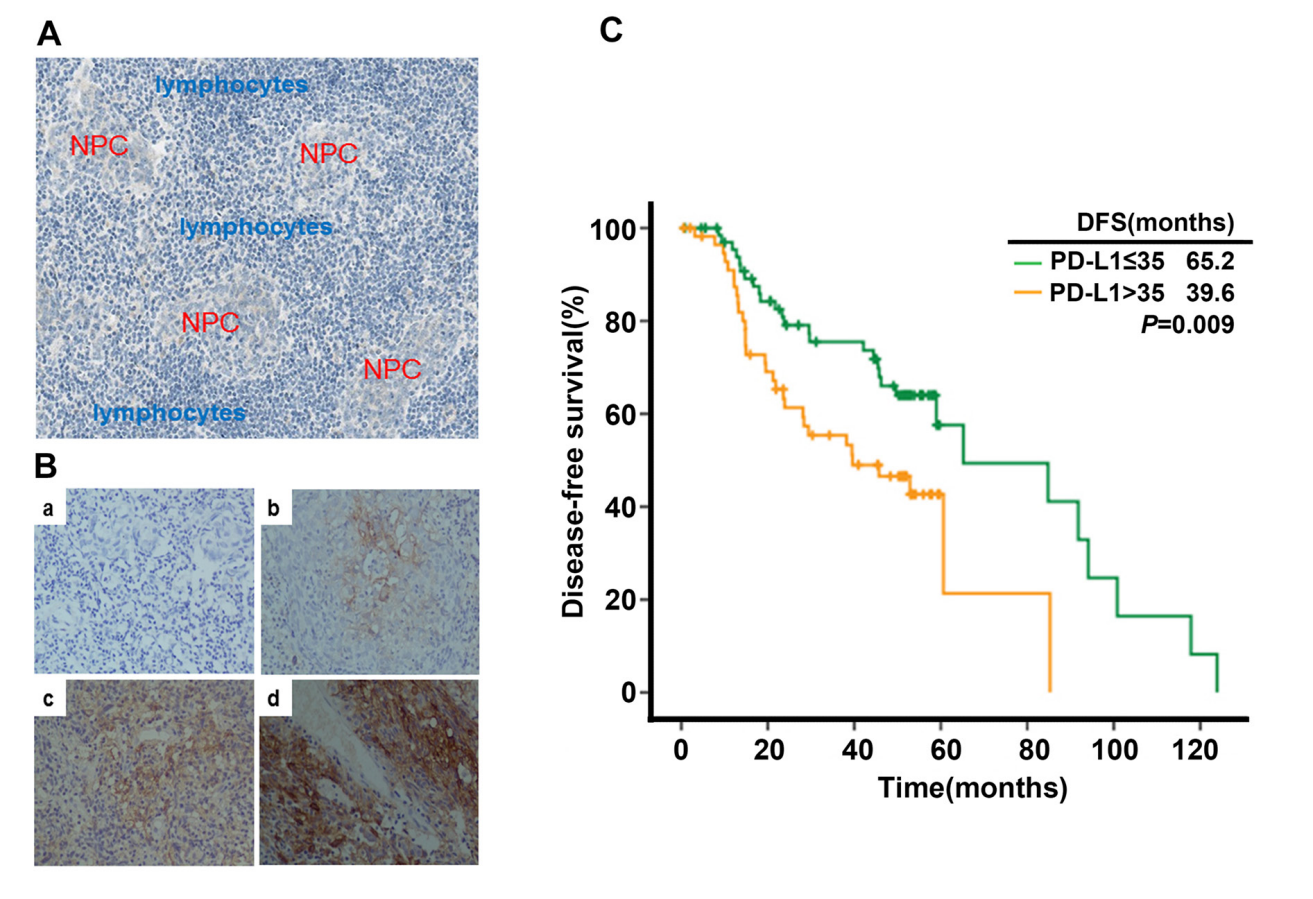
PD-L1 expression in tumor tissue samples and its correlation with recurrence free survival in nasopharyngeal carcinoma patients (A) Histological features of NPC: Nasopharyngeal carcinoma cells (red) were surrounded by infiltrating lymphocytes (blue). (B) PD-L1 expression in nasopharyngeal carcinoma sample (a. negative staining b. weak staining c. moderate staining d. strong staining). (C) Disease-free survival in nasopharyngeal carcinoma patients stratified by the expression level of PD-L1.

To determine the prognostic significance of PD-L1 in NPC, PD-L1 expression was analyzed with immunohistochemistry (IHC) method in 139 NPC samples. One representative Harris Hematoxylin and Eosin (HE) Staining of NPC nest was shown in Figure 6A. NPC cancer cells were surrounded by infiltrating lymphocytes (blue), which represents a distinct histological feature of NPC. We also tested the specificity of the employed anti-PD-L1 antibody for IHC. RT-PCR was utilized to detect PD-L1 mRNA in A549 and C666-1 cell lines using PD-L1-specific primers. There was no PD-L1 mRNA expression in A549 cell lines while high level of PD-L1 mRNA was detected in C666-1 cell lines (supplementary Figure S3-A). Then, we found the protein level of PD-L1 is undetectable in A549 cell line while C666-1 cell line has high level of PD-L1 protein by flow cytometry and IHC method (supplementary Figure S1-B, 1-C and 1-D). These results imply that the anti-PD-L1 antibody used in the present study is reliable for IHC research. Next we utilized IHC method to detect the expression level of PD-L1 in 139 NPC samples (Figure 6B,. negative staining b. weak staining c. moderate staining d. strong staining). Positive expression of PD-L1 (defined as more than 5% positively-stained cells). A total of 132 (95.0%) samples were determined to be PD-L1 positive. The baseline characteristics of all the 139 patients are shown in Table S1. Two groups with high (62/139; 44.6%) and low (77/139; 55.4%) PD-L1 expression were defined with cut-off value of H-score 35 (≤ 35 vs >35) by X-Tile. As shown in Table S2, the expression level of PD-L1 was not associated with clinical variables such as age, tumor stage, lymph node staging and clinical TNM staging. Univariate analysis showed that patients with high expression of PD-L1 (H-score >35) had poorer DFS compared with those with low PD-L1 expression (median DFS in H-score >35 vs H-score <35, 39.6 months vs 65.2 months, *P*=0.009) (Table S3, Figure 6C). Multivariate analysis demonstrated that PD-L1 was an independent prognostic factor for DFS in NPC patients (*P*=0.001, Table S4).

## DISCUSSION

NPC is one of EBV associated malignancies with high metastatic potency compared to other head and neck cancers, which is characterized by prevailing EBV infection and the presence of immune cell infiltration around tumor lesions [13-15, 25]. However, cancer cells could eventually evade immune elimination from host and keep growing, which indicates the existence of immunosuppressive microenvironment that makes these immune cells exhausted and anergic [5, 6, 26]. PD-L1 and PD1 are acknowledged as important immunosuppressive factors [6, 27]. Recently, PD-L1 was found to be up-regulated in some EBV-associated malignancies, including NPC [19]. However, the underlying mechanism of PD-L1 regulation and its clinical significance in EBV-associated NPC remains poorly understood.

In the present study, we found PD-L1 expression (both at protein and mRNA level) in NPC is positively associated with EBV infection. This result is similar with previous studies on some virus-associated cancers, such as EBV-associated lymphoma [19, 20], HBV-associated hepatocellular carcinoma [28], and HPV-positive head and neck squamous cell carcinoma [29, 30]. Other pathogen, such as Helicobacter pylori was also reported to induce PD-L1 expression in gastric cancer cell lines [31]. The phenomenon found here and in other studies implies that infection-associated cancers could create an “immune-privileged” milieu by up-regulating PD-L1. Therefore, targeting the PD-L1/PD1 pathway to break off immune suppression confers promising anti-neoplastic strategy for infection-associated cancers. The underlying mechanisms of PD-L1 up-regulation in EBV-infected NPC were further investigated in the present study.

Previous studies demonstrated that constitutive oncogenic pathway could up-regulate PD-L1 (innate immune resistance) [6]. Parsa AT et, al found loss of phosphatase and tensin homolog (PTEN) and the resulting activation of phosphatidylinositol-3-OH kinase (PI-3K) pathway significantly elevates PD-L1 expression in glioma [32]. Similarly, constitutive activation of NPM/ALK was reported to drive PD-L1 expression through STAT3 [33]. Oncogenic EGFR signaling pathway in non-small-cell lung cancer (NSCLC) may trigger the expression of PD-L1 and hence immune resistance [34]. In addition, EML4-ALK rearrangement in NSCLC was also found to provoke PD-L1 expression in our ongoing experiments (un-published data). Therefore, we sought to investigate whether LMP1, a well-recognized initiator of oncogenic pathway in EBV-infected NPC [23, 24, 35], participates in PD-L1 regulation. As previously reported, LMP1remarkably increased the activity of STAT3, NF-κ B, and AP-1, altering the expression of critical proteins involved in the proliferation, anti-apoptosis, and invasion of cells and ultimately leading to tumorigenesis [22-24, 35]. In the current study, we found the expression of PD-L1 in EBV positive cell lines was positively related to LMP1 (Figure 2). Furthermore, we found either exogenous over-expression of LMP1 or endogenously induced LMP1 expression by TPA could significantly increase PD-L1 expression (Figure 3). The up-regulation of PD-L1 mediated by LMP1 was associated with the activation of STAT3, AP-1, and NF-κ B. By inhibiting phosphorylated JAK3, the level of PD-L1 decreased following pSTAT3 down-regulation in a clear dose-response manner, indicating that JAK3-STAT3 pathway plays a critical role in remodeling the expression of PD-L1 by LMP1. ERK1/2/AP-1 and NF-κ B pathways were also found to participant in the regulation of PD-L1. The above-mentioned pathways were further validated in C666-1 (a NPC cell line constitutively carrying EBV). These results show that the constitutive oncogenic pathways mediated by LMP1 are at least partially responsible for the up-regulation of PD-L1 in EBV positive NPC. This previously undefined function of LMP1 may provide new insights into the immune escape and tumorigenesis of EBV-driven NPC.

**Figure 7:**
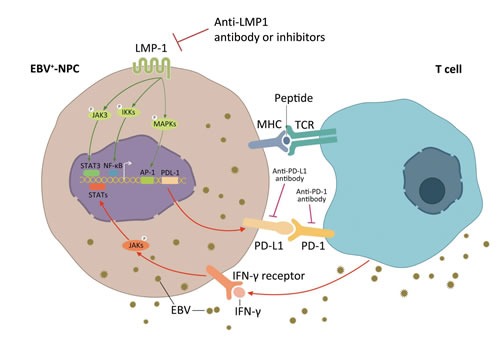
Two mechanisms of up-regulated PD-L1 expression on EBV positive nasopharyngeal carcinoma cells PD-L1 is a well-known immune suppressive factor in a variety of cancer types. Two possible mechanisms of PDL1 regulation in EBV positive NPC was proposed. The first one (innate immune resistance): constitutive oncogenic pathway activation mediated by LMP1 up-regulates PD-L1 expression, which is independent of inflammatory signals in the tumor microenvironment; and the second one (adaptive immune resistance): PD-L1 is induced in response to inflammatory signals, such as IFN-γ, which are produced during an active anti-viral and anti-tumor immune response.

Apart from the innate immune resistance mediated by LMP1 in EBV positive NPC, an alternative mechanism of PD-L1 up-regulation was also found in the present study. Previous studies have found that many inflammatory factors are up-regulated through the antitumor and/or antiviral immune response, which may be utilized by cancer cell itself to evade immune surveillance [6, 36, 37]. Among these inflammatory factors, IFN-γ was the most recognized one in modulating PD-L1 expression [6, 38]. IFN-γ can regulate PD-L1 at transcription level by initiating the synthesis of interferon regulatory factor-1 (IRF-1), a transcriptional factor which has two binding sites on PD-L1 promoter, through JAK/STAT pathway [39]. Another post-transcriptional mechanism of regulating PD-L1 expression involves miR-513, which is complementary to the PD-L1 3′-UTR. IFN-γ treatment decreases miR-513 level and hence the up-regulation of PD-L1 mRNA [40]. Indeed, we found the level of serum IFN-γ was positively related to EBV burden in NPC patients. IFN-γ remarkably increased the expression of PD-L1 independent of LMP1 in NPC cell lines. Interestingly, LPM1+ NPC cell lines treated with IFN-γ were found to have higher level of PD-L1 expression compared with LMP1- cell lines (Figure 5B). These results imply that the innate immune resistance mediated by LMP1 oncogenic pathways and the adaptive immune resistance in response to inflammatory signals like IFN-γ are two distinct but synergistic mechanisms of PD-L1 regulation in EBV positive NPC. These two critical mechanisms of up-regulating PD-L1 expression in EBV-related NPC are proposed in Figure 7.

We finally evaluate the prognostic value of PD-L1 for EBV-infected NPC. We found that lower PD-L1 level was correlated with a significantly longer disease-free survival in NPC patients, indicating PD-L1 is a poor prognostic factor in NPC (Figure 6). However, the clinical significance of PD-L1 status in various tumors has not been definitely established. Zeng Z et al found that circulating PD-L1 could serve as an independent predictor of overall survival and tumor-recurrence survival in HCC patients after cryoablation [41]. In ovarian cancer, the expression of PD-L1 on tumor cells is independently associated with poorer progression-free survival and overall survival [42]. Other cancer types, including renal cell carcinoma, gastric cancer, and pancreatic cancer also show PD-L1 as a poor prognostic factor [43-45]. However, more recent studies found PD-L1 was a better prognostic factor in melanoma [36], colorectal cancer [46], Merkel cell carcinoma [47] and non-small-cell lung cancer [48]. The discrepancy across different studies may be due to variations in IHC technique, cancer type, stage of cancer analyzed and treatment history. In our study, PD-L1 was found to be regulated by both LMP1 oncogenic pathway and inflammator signals such as IFN-γ. Therefore, PD-L1 may represent LMP1 mediated tumorigenesis, immune escape as well as host's antitumor immune response. The different clinical significance of PD-L1 may be determined by its predominant regulator mechanism (oncogenic pathway mediated innate immune resistance or adaptive immune resistance during antitumor response). One limitation of the present study is that it was an in vitro study. Therefore, using orthotopic mouse model to assess the efficacy of anti-PD-L1/PD-1 and/or anti-LMP1 therapy in vivo is of significance for pre-clinical studies [49].

In conclusion, EBV-infected NPC has higher level of PD-L1 expression at least through LMP1 mediated oncogenic pathways and immune modulation through the excretion of IFN-γ. Lower PD-L1 level is associated with better local disease control. To our knowledge, this is first study to explore the detailed mechanism of PD-L1 up-regulation in NPC with EBV infection. Our results highlight the potential clinical benefits of blocking both LMP1 oncogenic pathway and PD-1/PD-L1 check points in treating EBV-infected NPC patients.

## MATERIALS AND METHODS

### Cell lines and cell culture

Human NPC cell line 6-10B, SUNE-1, 5-8F, CNE-1, CNE-2, TWO3, HNE-1 and EBV-positive NPC cell line C666-1 were routinely kept in Sun Yat-Sen University Cancer Center (Guangzhou, China). TWO3-EBV-, TWO3-EBV+ cells and were kindly provided by Dr. Li Jiang (Sun Yat-Sen University Cancer Center, Guangzhou, China). CNE-2-EBV^−^, CNE-2-EBV^+^ cells and stable cell lines NP-69-vector, NP-69-LMP1 were nicely provided by Prof. Zeng Musheng (Sun Yat-Sen University Cancer Center, Guangzhou, China). Stable cell lines CEN-2-vector and CNE-2-LMP1 were kindly provided by Prof. Huang Bijun (Sun Yat-Sen University Cancer Center, Guangzhou, China). All NPC cells were incubated in RPMI-1640 medium supplemented 10% fetal bovine serum and antibiotics (10000 U/ml penicillin and 10μg/ml streptomycin). The immortalized nasopharyngeal epithelial cell line NP-69 [50] and its constructed NP-69-vector, NP-69-LMP1 stable cell lines was cultured in keratinocyte serum-free medium (Invitrogen, Carlsbad, CA) supplemented with 25 mg/ml bovine pituitary extract, and 0.2 ng/ml recombinant epidermal growth factor per manufacturer's instructions. All cells were maintained in a humidified incubator at 37°C with 5% CO^2^.

### RNA extraction and PCR

To quantify PD-L1 mRNA expression, total RNA was isolated and cDNA was synthesized using TaqMan MultiScribe Reverse Transcriptase (Applied Biosystems, FosterCity, CA) as previously described [51]. Quantitative real-time PCR analysis was performed using an ABI Prism 7900-HT Sequence Detection System (96-well, AppliedBiosystems) and Semi-quantitative PCR was performed using Bio-Rad MyCycler PCR System. Primers for this study included: forward primer 5#-CCTACTGGCATTTGCTGAACGCAT-3# and reverse primer 5#-ACCATAGCTGATCATGCAGCGGTA -3# for PD-L1; forward primer 5#-CTCTTGGCTGTTACTGCCAGG-3# and reverse primer 5#-CTCCACACTCTTTTGGATGCT-3# for IFN-γ. Primers used for β-actin were previously reported [51]. The total semi-quantitative PCR product was then run on a 2% agarose gel.

### Western blot analysis

Cells were harvested and suspended in RIPA lysis buffer (Thermo, Hercules, CA) containing a protease inhibitor cocktail (Sigma–Aldrich Corporation, St Louis, MO). After incubation on ice for 15 min, cell lysates were centrifuged at 13 000 r.p.m. for 15 min at 4°C. The protein content of the supernatant was determined using the Thermo Protein Assay Reagent (Thermo, Hercules, CA). 30-60 μg Proteinsper well were separated by 12% sodiumdodecyl sulfate–polyacrylamide gel electrophoresis and transferred topolyvinylidene difluoride membranes (Bio-Rad Laboratories). The following primary antibodies were used to probe the alterations of protein:LMP1(CS1-4, Dako), PD-L1(E1L3N™, Cell Signaling Technology, Danvers, MA), p-stat3, total-stat3, p-NF-κB (P65), NF-κB (P65), p-c-fos, p-c-Jun, p-JAK3, JAK3, p-ERK1/2, ERK1/2 (Cell Signaling Technology, Danvers, MA) and β-actin (Santa Cruz Biotechnology, Santa Cruz, CA). Signals were detected by enhanced chemiluminescence Plus reagents (Amersham Pharmacia, Piscataway, NJ). Signal quantification was obtained using Quantity One software (Bio-Rad Laboratories, Hercules, CA) and normalized to β-actin.

### Immunofluorescence

Human SUNE-1, C666-1, TWO3-EBV^−^, TWO3-EBV^+^, CNE-2-EBV^−^, CNE-2-EBV^+^ cells grown on a chamber slide(BD Biosciences, San Jose, CA) were washed with cold PBS, fixed with 4% paraformaldehyde in phosphate-buffered saline (PBS) for 10 min. After 1h blocking in PBS + 0.1% Tween-20 plus 3% donkey serum, cells were incubated with primary antibodies of PD-L1 (E1L3N™, Cell Signaling Technology, Danvers, MA) at 4°C overnight, after three times of washing, then with secondary antibody (Alexa Fluor 555 donkey anti-rabbit IgG (H+L), Life Technologies, LA) for 1 h at room temperature. After counterstaining with DAPI (1 μg/ml) for 10 min, slides were observed and photographed with fluorescence microscopy. These experiments were triplicated.

### Surface staining for flow cytometry

Attached cells were digested with 0.2% trypsin with 0.25% EDTA. Suspending cells (10^6^) were fixed with 4% formaldehydein PBS for 10 minutes. After washing, pre-incubated with 3% donkey serum in PBS for 30 min in ice, cells were stained with the PD-L1 antibody (E1L3N™) or with the proper isotype control lgG for 45 min in ice. Cells were washed twice with PBS by centrifugation. Anti-rabbit IgG (H+L), F(ab')2 Fragment (Alexa Fluor® 647 Conjugate, #4414, Life Technologies, LA) was used as a secondary antibody. After washing, cells were analyzed by flow cytometry on a FACScan (BD, Biosciences) instrument. The data were analyzed with the Cell Quest program (BD Biosciences) and WinMDI software.

### Transient transfection

Briefly, 4 × 10^5^ cells of CNE-2 and TWO3 per well were plated into six-well plates and grown for one day in antibiotic-free medium containing 10% PBS prior to transfection. Plasmid pZip-NeoSV-LMP1and control vector Plasmids was provided by Prof. Zeng Musheng (Sun Yat-Sen University Cancer Center, Guangzhou, China) were performed with Lipofectamine 2000 (Invitrogen, CA) according to the manufacturer's instructions. Further assays were conducted after 48h incubation of transiently transfected cells.

### Small interfering RNA experiments

The LMP1 and negative control siRNA were chemically synthesized by Ribo Bio, Co, Ltd (Guangzhou, China). The sequences of LMP1 siRNA (EU000388, miRNA nucleotide 371-389) were: sense sequence, 5′-GGA AUU UGC ACG GAC AGG CTT-3′; anti-sense sequence, 5′-GCC UGU CCG UGC AAA UUC CTT-3′ and the sequences of negative control siRNA were: sense sequence, 5′-UUC UCC GAA CGU GUC ACGUTT-3′; anti-sense sequence, 5′-ACG UGA CAC GUUCGG AGA ATT-3′ as previously described [52]. Cells were seeded in a 6-well plate with 2×10^5^ cells per well in growth medium without antibiotics. The transfections in our study were performed with RNAi MAX Transfection Reagent (Invitrogen) according to the manufacturer's protocols.

### 12-O-tetradecanoyl phorbol 13-acetate (TPA) and Inhibitors treatment

For 12-O-tetradecanoyl phorbol 13-acetate (TPA) treatment, CNE-2-EBV^+^ and TWO3-EBV^+^ cells were treated with 50ng/ml 12-O-tetradecanoyl phorbol 13-acetate (TPA, Sigma–Aldrich Corporation, St Louis, MO) for 0, 12, 24 or 48 hours. Cells were harvested for western blot analysis. For inhibitors treatment, NP-69 and NP-69-LMP1 and C666-1 cells were first serum-starved for 6h and then treated with growth medium with 0.01% DMSO plus different concentrations of highly selective JAK3 inhibitor (Tofacitinib, CP-690550, Selleckchem), MEK inhibitor (PD0325901, Selleckchem) or NF-κB inhibitor (Caffeic Acid Phenethyl Ester, Selleckchem) for another 72h. Cells were harvested for protein alteration by western blot.

### Quantification of EBV-DNA copy number

A 5-mL peripheral blood of patients was obtained. Plasma was isolated by centrifuging at 2000 r.p.m for 10 minutes. DNA was extracted from 200 μL of plasma, using QIAamp DNA blood kits (Qiagen K.K.). A real-time quantitative PCR assay was carried out and the result was expressed as copies per 1 mL of sample, as previously described [53].

### IFN-γ analysis by ELISA

2-3 ml peripheral blood from patients was obtained. Serum was isolated by centrifuging at 2000 r.p.m for 10 minutes. Peripheral blood mononuclear cells (PBMCs) were isolated from 30 ml heparinized blood from healthy donors by Ficoll/Isopaque gradient fractionation. PBMCs were stimulated with phorbol12-myristate13-acetate (PMA) and ionomycin for 6 hours. Activated PBMCs were cultured in 10% RIPM medium for 48h. Cell growth medium was harvested by centrifuging at 2000 r.p.m for 10 minutes. PBMCs growth medium was used as positive control and cell-free growth medium was used as negative control for IFN-γ production analysis. IFN-γ level in serum and cell growth medium was determined using ELISA kit Bio-Plex Pro™ (Bio-Rad Laboratories, Hercules, CA, USA) per manufacturer's protocol.

### Immunohistochemistry

4–5-μm formalin-fixed paraffin embedded tissue (FFPE) of human NPC tissue, A549 add C666-1 cells specimen were deparaffinized, rehydrated, and quenched with 1.5% H_2_O_2_. For antigen retrieval, slides were treated with Dako Cytomation Target Retrieval Solution (Dako, Carpinteria, CA) in a steam bath at 95 °C for 45 min. After equilibration in PBS for15 min, slides were placed in an auto stainer apparatus (Dako) and incubated with anti-PD-L1 antibody (E1L3N™, Cell Signaling Technology, Danvers, MA) at 1:200 dilution at room temperature for 30 min. Immunoreactivity was detected using the Dako EnVision method according to the manufacturer's instructions. For negative controls, slides were subjected to the same procedure, including antigen retrieval, except for omission of the primary antibody. The results were reviewed independently by 2 surgical pathologists, who were blinded to the clinical or pathological information of these patients. A semi-quantitative scale from 0 to 100% was used to grade (0~+++) of PD-L1 stained cancer cells and mesenchymal cells. The average score of replicate samples was used in the subsequent analyses.

### Patients and clinical data

Two cohorts of patients with NPC were enrolled into the research. All patients were treated in Sun Yat-Sen University Cancer Center (Guangzhou, China) from 1 January 2004 to 31 August 2008.

The first cohort consisted of 34 consecutive NPC patients. Baseline plasmid and pre-treatment serum was collected for EBV-DNA copy number and plasmid IFN-γ level analysis as described in materials and methods.

The second cohort included 139 adult patients diagnosed of NPC in Sun Yat-Sen University Cancer Center (Guangzhou, China), who had FFPE from the original diagnostic biopsy, were identified. The basic clinical data of these patients were collected, including gender, age, tumor stage, treatment regimen and follow-up records.

Characteristics of these patients are summarized in table 1S. Among the 139 patients enrolled, 113 males and 26 females, with the median age 45 years (range from 18 to 81 years). All the patients were treated with conventional chemo-radiotherapy. The median follow-up time was 50.3 months. Locoregional relapse or distant metastasis had occurred in 60 patients and a total of 30 patients had died during follow-up. All tumors were classified as undifferentiated non-keratinizing phenotype. Among this tissues, 110/139 (79%) are available for Epstein-Barr virus encoded RNAs (EBERs) hybridization analysis.108/110 (98%) tissues were EBERs positive. Among all patients, 40 cases' plasma EBV burden was tested. The plasma EBV burden ranged from 100 to 6.8×10^6^ copies per ml.

The study protocol was approved by the Institutional Review Board of Sun Yat-Sen University Cancer Center (Guangzhou, China) and was conducted in accordance with the Declaration of Helsinki and good clinical practice. All the patients had provided written informed consent before samples were collected.

### Statistical analysis

For experimental part, numerical data are presented as the mean ± standard deviation of the mean (SD). A standard two-tailed Student's t-test and a paired Student's t-test were used for comparison of the numerical data, and *P*-values less than 0.05 were considered significant.

Patients were divided into high and low PD-L1 expression groups. Optimal cut-off point for PD-L1 was determined by using the X-Tile statistical package (Yale University, New Haven, CT) based on the outcome [54]. Kaplan-Meier curve defined by this cut point was generated, and statistical significance of difference arising from differential expression of PD-L1 was determined by using the log-rank test. Disease-free survival (DFS) was measured from the date of therapy accomplished to the time of recurrence, metastasis or the date of last follow-up. Student's t-test was used to evaluate the association of high and low expression of PD-L1 with age. Chi-square test was used to assess the expression of PD-L1 with clinical parameters such as gender and tumor staging. Survival analysis was depicted by Kaplan-Meier method. Univariate analysis and multivariate analysis were performed with log-rank test and Cox regression analysis, respectively. A *p* value of <0.05 used to denote statistical significant, and all reported *p* values were two sided. These statistical analyses were performed with SPSS 20.0 (Chicago, IL, USA).

## SUPPLEMENTARY MATERIAL, FIGURES AND TABLES


