# Supplementary material for: EBV-driven LMP1 and IFN-γ up-regulate PD-L1 in nasopharyngeal carcinoma: Implications for oncotargeted therapy
Source: Oncotarget. 2014 Oct 21;5(23):12189–202. doi: 10.18632/oncotarget.2608 (PMC4322961; doi:10.18632/oncotarget.2608)
Supplement: Supplementary file 1 [file oncotarget-05-12189-s001.pdf]

EBV-driven LMP1 and IFN-γ up-regulate PD-L1 in nasopharyngeal carcinoma: Implications for oncotargeted therapy

Supplementary Material

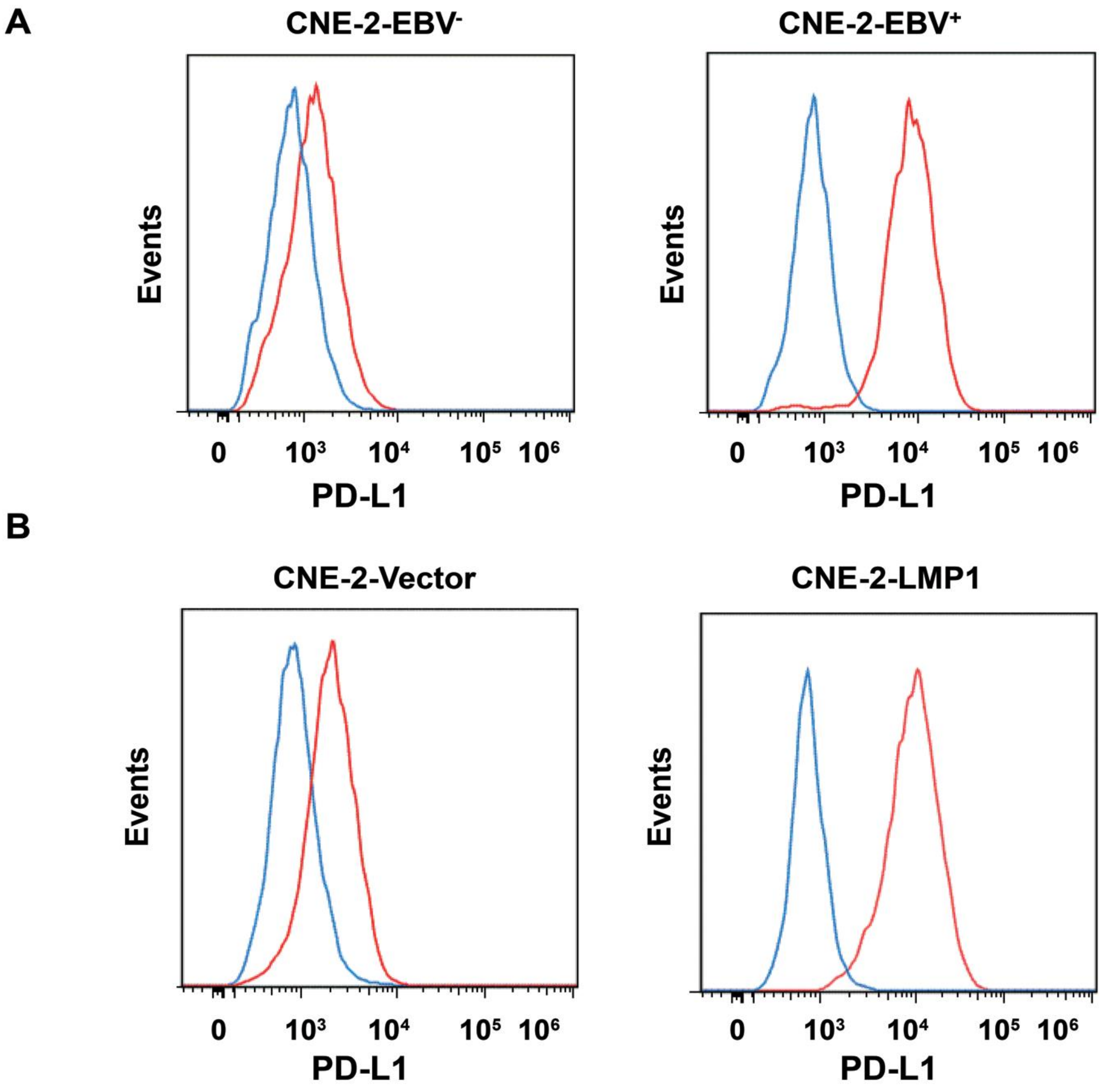

Supplementary Figure S1: PD-L1 expression is induced by EBV infection and LMP1 over-expression. (A) PD-L1 expression in CNE-2 and CNE-2-EBV^{+} cells was analyzed with flow cytometry. (B) PD-L1 expression in CNE-2-Vector and CNE-2-LMP1 cells was analyzed with flow cytometry. All experiments were repeated at least three times. Representative data are shown.

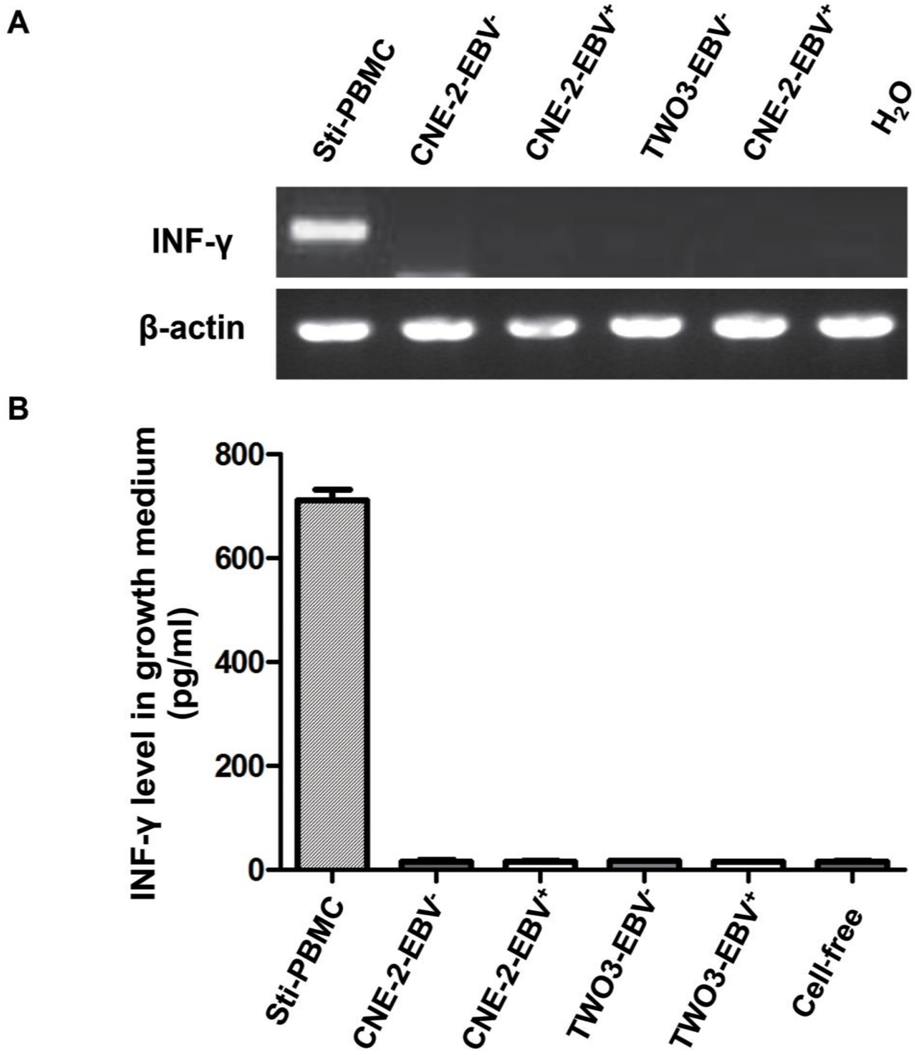

Supplementary Figure S2: EBV infection does not affect the INF-γ production in human NPC cells. (A) Relative quantitative analysis PD-L1 mRNA level in CNE-2, CNE-EBV$^+$ cells and TWO3, TWO3-EBV$^+$ cells (phorbol12-myristate13-acetate (PMA) and ionomycin stimulated human PBMCs cells were used as positive control and nucleic acid free H_2O was used as negative control). (B) IFN-γ level was measured in CNE-2, TWO3 cells and EBV infected CNE-2, TWO3 cells growth medium (phorbol12-myristate13-acetate (PMA) and ionomycin stimulated human PBMCs growth medium was used as positive control and cell-free culture medium was used as negative control).

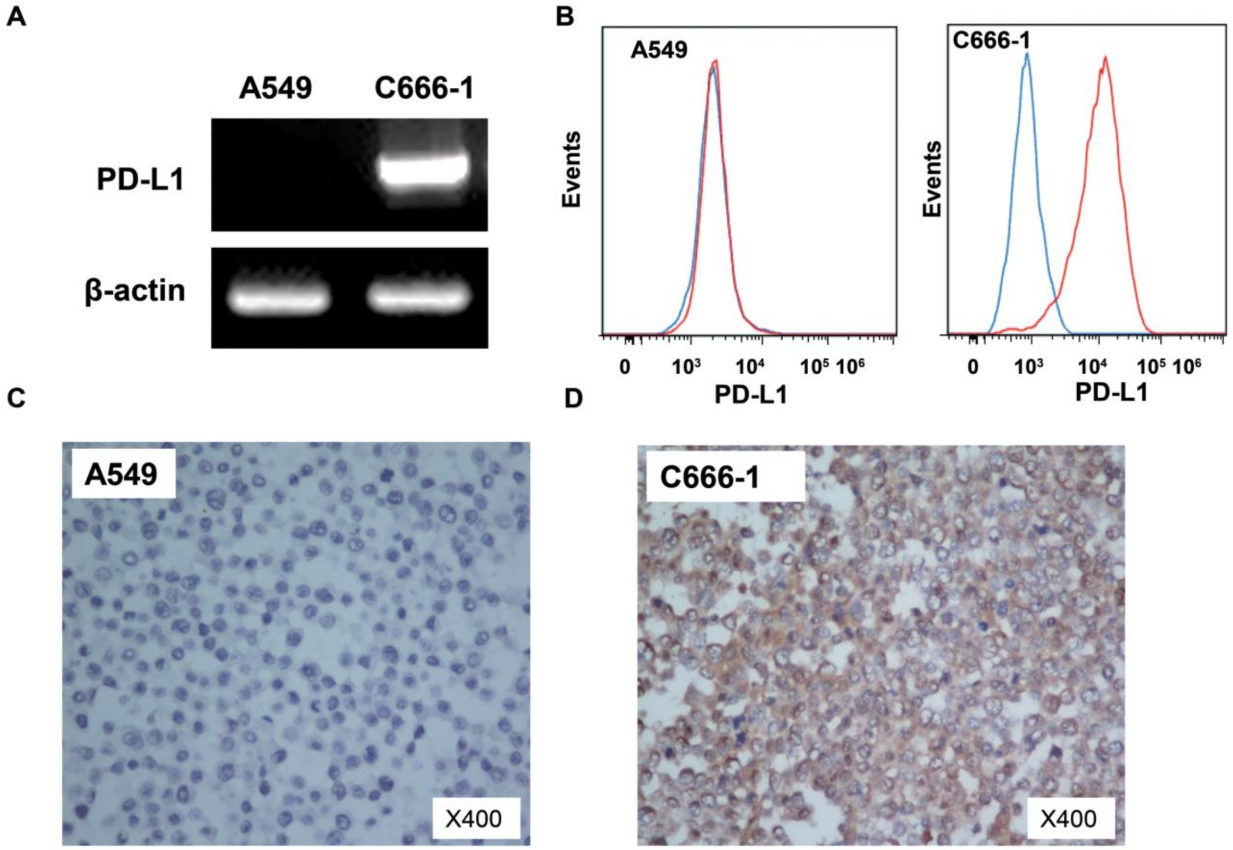

Supplementary Figure S3: The specificity validation of the PD-L1 antibody. (A) Relative quantification of PD-L1 mRNA level in A549 cells and C666-1 cells was analyzed with RT-PCR. β-actin was used to verify equal loading.(B) Cell-surface PD-L1 expression in A549 and C666-1 cell lines was analyzed with flow cytometric analysis (PD-L1, red line; isotype controls, blue line). (C, D) PD-L1 expression in A549 and C666-1 was analyzed with IHC. All experiments were repeated at least three times. Representative data are shown.

Table S1: Baseline clinical characteristic of all patients.

Characteristics	(n=139)	
	Cases (n)	Percentage (%)
**Age (years)**		
Median (range)	45 (18-81)	
**Gender**		
Male	113	81.3
Female	26	18.7
**Staging**		
I	2	1.4
II	26	18.7
III	59	42.4
IVa	28	20.1
IVb	12	8.6
IVc	12	8.6
**Recurrence or metastasis***		
Yes	60	47.2
No	67	52.8

* Patients diagnosed at Stage IVc were excluded.

Table S2: Correlation between clinical parameters and the expression of PD-L1 in biopsies of nasopharyngeal carcinoma.

Parameter	PD-L1 expression		
	**PD-L1(h), n=62**	**PD-L1(l), n=77**	***p***
**Age**	44.1±12.5	46.5±12.0	0.83
**Gender**			
Male	52 (82.5)	61 (80.3)	0.73
Female	11 (17.5)	15 (19.7)	
**Tumor stage**			
T1	3 (4.8)	1 (1.3)	0.42
T2	25 (39.7)	24 (31.6)	
T3	22 (34.9)	32 (42.1)	
T4	13 (2.1)	19 (25.0)	
**Nodal status**			
N0	15 (23.8)	11 (14.5)	0.12
N1	28 (44.4)	26 (34.2)	
N2	15 (23.8)	27 (35.5)	
N3	5 (7.9)	12 (15.8)	
**TNM stage**			
I	2 (3.2)	0	0.052
II	15 (23.8)	11 (14.5)	
III	29 (46.0)	30 (39.5)	
IV	17 (27.0)	35 (46.1)	

Abbreviation: PD-L1(h), high expression (H-score >35) of PD-L1; PD-L1(l), low expression (H-score≤ 35) of PD-L1;

Table S3: Univariate analysis to assess the association of clinical parameters with prognosis of nasopharyngeal carcinoma.

**Items**	Cases (n)	Univariate analysis P^a **DFS**
**Age (years)**		
≤ 45	71	0.88
> 45	68	
**Gender**		
Male	113	0.52
Female	26	
**TNM stage**		
Stage I, II	28	0.73
Stage III, IV	111	
**PD-L1**		
Low (H-score ≤ 35)	77	**0.009**
High (H-score > 35)	62	

Note: [a] Log-rank test.

Abbreviations: PD-L1, programmed death- ligand 1; DFS, disease-free survival.

Table S4: Multivariate analysis for PD-L1 as a prognostic factor of nasopharyngeal carcinoma.

Items	n	DFS	
		HR (95%CI)	*p* value
**PD-L1**			
Low (H-score ≤ 35)	77	2.78 (1.53-5.03)	**0.001**
High (H-score > 35)	62		

Abbreviation: PD-L1, program death-ligand 1; DFS, disease-free survival.
